# Prediction of Regulatory SNPs in Putative Minor Genes of the Neuro-Cardiovascular Variant in Fabry Reveals Insights into Autophagy/Apoptosis and Fibrosis

**DOI:** 10.3390/biology11091287

**Published:** 2022-08-30

**Authors:** Andrea Virginia Ruiz Ramírez, Ernesto Prado Montes de Oca, Luis E Figuera

**Affiliations:** 1 Doctorado en Genética Humana, Centro Universitario de Ciencias de la Salud (CUCS), Universidad de Gudalajara, Sierra Mojada No. 950, Col. Independencia, Guadalajara 45180, Jalisco, Mexico; 2 División de Genética, CIBO-IMSS, Sierra Mojada No. 800, Colonia Independencia, Guadalajara 45180, Jalisco, Mexico; 3 Laboratorio Nacional de Medicina Personalizada (LAMPER), Biotecnología Médica y Farmacéutica, Unidad Guadalajara, CIATEJ AC, Consejo Nacional de Ciencia y Tecnología (CONACYT) Av. No. 800, Colinas de la Normal, Guadalajara 45180, Jalisco, Mexico

**Keywords:** Fabry disease, promoter, regulatory SNPs, modifying genes, clinical variability, *IL10*, TGFB1, EDN1

## Abstract

**Simple Summary:**

Even though in monogenic diseases a mutation will lead to a “classic” manifestation, many disorders exhibit great clinical variability that could be due to modifying genes also called minor genes. Fabry disease (FD) is an X-linked inborn error resulting from the deficient or absent activity of alpha-galactosidase A (α-GAL) enzyme, that leads to deposits of globotriaosylceramide. With our proprietary software SNPclinic v.1.0, we analyzed 110 single nucleotide polymorphisms (SNPs) in the proximal promoter of 14 genes that could be modifying the phenotype of FD. We found seven regulatory-SNP (rSNPs) in three genes (*IL10*, *TGFB1* and *EDN1*) in five cell lines relevant to FD (cardiac myocytes, cardiac fibroblasts, astrocytes-cerebellar, endothelial cells and T helper cells 1-T_H_1). Each SNP was confirmed as a true rSNP in public eQTL databases and prediction of variants was suggested by additional software. The two proposed rSNPs in *IL10*, could explain components for the regulation of active B cells that influence the fibrosis process. The three predicted rSNPs in *TGFB1*, could act in apoptosis-autophagy regulation. The two putative rSNPs in *EDN1*, putatively regulate chronic inflammation. The rSNPs described here could act to modulate Fabry’s clinical phenotype so we propose that *IL10*, *TGFB1* and *EDN1* be considered genetic modifiers in FD.

**Abstract:**

Even though a mutation in monogenic diseases leads to a “classic” manifestation, many disorders exhibit great clinical variability that could be due to modifying genes also called minor genes. Fabry disease (FD) is an X-linked inborn error resulting from the deficient or absent activity of alpha-galactosidase A (α-GAL) enzyme, that leads to deposits of globotriaosylceramide. With our proprietary software SNPclinic v.1.0, we analyzed 110 single nucleotide polymorphisms (SNPs) in the proximal promoter of 14 genes that could modify the FD phenotype FD. We found seven regulatory-SNP (rSNPs) in three genes (*IL10*, *TGFB1* and *EDN1*) in five cell lines relevant to FD (Cardiac myocytes and fibroblasts, Astrocytes-cerebellar, endothelial cells and T helper cells 1-T_H_1). Each SNP was confirmed as a true rSNP in public eQTL databases, and additional software suggested the prediction of variants. The two proposed rSNPs in *IL10*, could explain components for the regulation of active B cells that influence the fibrosis process. The three predicted rSNPs in *TGFB1*, could act in apoptosis-autophagy regulation. The two putative rSNPs in *EDN1*, putatively regulate chronic inflammation. The seven rSNPs described here could act to modulate Fabry’s clinical phenotype so we propose that *IL10*, *TGFB1* and *EDN1* be considered minor genes in FD.

## 1. Introduction

In monogenic diseases a mutation leads to a “classic” manifestation, but many disorders exhibit a great clinical or phenotypical variability that cannot be explained only by the mutation in the major gene. This variability could be due to modifying or minor genes that modulate the disease’s phenotype [1]. Such genes could act directly on the gene product causing the disease or indirectly on alternative etiopathogenic pathways [2]. Therefore, the role those genetic variants play in each disease can be in the phenotypic expression, severity, or even the age of onset in patients [3]. When genetic interactions occur, such as combinations between mutations in different genes, an unexpected phenotype may occur, which would differ from the effects of the individual mutant phenotypes [4]. The effect of the combination of mutations has been studied in the cardiomyopathy called left ventricular non-compaction (LVNC). In a family of three children with early-onset cardiomyopathy, the complete exome was sequenced and the presence of three variants in the *MKL2*, *MYH7* and *NKX2* genes was found, the variants in the first two genes mentioned were inherited from the asymptomatic affected father and the rare variant in NKX2 from the unaffected mother. The evaluation of functional consequences in vivo in murine models led to the conclusion that NKX2 acted as a modifier gene [5]. It is important to emphasize that the effect may not only be towards an increase in clinical features but also a suppression effect by interaction can also occur, where some modifying gene may suppress the expected phenotype [6]. Both effects have been seen in rare Mendelian diseases, such as lysosomal diseases, but much remains to be deciphered.

Fabry disease (FD) (OMIM #301500) [7] is an X-linked inborn error of glycosphingolipid catabolism resulting from the deficient or absent activity of the lysosomal enzyme alpha-galactosidase A (α-GAL). The functional modification of the enzyme causes the accumulation of complex sphingolipids, especially globotriaosylceramide (Gb3) [8].

Depending on the enzyme activity, two main phenotypes in FD have been recognized: the multisystemic subtype with a classic phenotype since childhood due to scarce or null α-GAL activity, and the late-onset subtype which usually begins in adulthood with failure of organs such as the heart, kidney or brain and results from the partial activity of α-GAL [9].

Vascular endothelium cells are the main affected, it has been observed that excessive intracellular Gb3 induces oxidative stress and expression of cell adhesion molecules in endothelial cells [10]. The exact location of these deposits has been identified in GLA KO mouse [11] in which the accumulation of Gb3 occurs in the lipid rafts of the cell membranes [12]. The deposits lead to pathogenic cascades that culminate in an inflammatory response in any tissue [13]. This complex process requires the participation of inflammatory cytokines (IFNγ, TNF-α), chemokines, effector enzymes as well as anti-inflammatory cytokines (IL-10, IL-4, and IL-13) that counteract any chronic inflammation. A dysfunctional autophagy pathway that could contribute to the pathological process has been found in cultured renal cells, fibroblasts, and lymphocytes of patients with FD [14]. The presence of cytokine IL-10, highly expressed in FD is also a negative regulator of autophagy [15,16].

Fibrosis has been found in the organs mainly affected by FD, at the renal level it has been seen that the progression goes from podocyte injury to the generation of fibrosis, at the cardiac level, fibrosis can be found even in the early stages of cardiomyopathy [17]. In studies with a fibrosis-mimicking device, when TGF-β1 is administered to fibroblasts, it induces a change in several cytokines and reflects the fibrotic process, accompanied by cellular responses such as epithelial-mesenchymal transition (EMT) [18].

Another key component that can directly affect both renal and cardiac levels is vascular function. Rohard et al. [19] analyzed polymorphisms in *NOS3*, a gene with the function of producing nitric oxide (NO), finding that the genotypes of this gene could partly explain the variability of cardiac phenotypes in FD.

For this reason, we decided to analyze in this article genes related to interleukins, fibrosis-sclerosis, renal disease and endothelial or vascular disease that could be modifying the clinical characteristics of FD, to find, through our proprietary software SNPclinic, regulatory variants with clinical significance.

## 2. Materials and Methods

### 2.1. Input for the SNPClinic v.1.0

For this study, 14 genes were selected, related either directly to the injury of an organ affected by FD or indirectly, these were selected through PubMed [20]. By using our own SNPClinic software v.1.0 [21], we first generated in silico human proximal (2 Kb from transcriptional start site) pseudo-promoters comprising all common SNPs (MAF > 1%) in the averaged world population (code = ALL) according to the 1000 Genomes Project for each of the 14 modifying genes in FD (Table 1). Subsequently, to assess chromatin accessibility, we obtained DNAseI- HUP data from five FD/ modulator genes cell lines from ENCODE project, namely Hcm (human cardiac myocytes), Hcf (primary human cardiac fibroblasts), Hac (astrocytes-cerebellar), Huvec (umbilical vein endothelial cells), T_H_1 (Immunophenotype of T-helper 1 lymphocytes).

Transcription factor Position Frequency Matrices (PFMs) were obtained from the JASPAR [22] database. Finally in this step, we obtained chromosome coordinates, biallelic alleles, DNAse-HUP accessibilities, and PFMs as input data.

### 2.2. Prediction of Regulatory SNPs (rSNPs) with SNPclinic v.1.0

We followed the method of Flores Saiffe et al. [21], where SNPClinic scans base-per-base the proximal promoter for each gene for each of the two DNA strands calculating the local DNA affinity to each of the 396 human transcription factors from the JASPAR Database. In this way, SNPClinic explores the overlap of chromatin accessibility between the DNase HUP-1 of the cell lines mentioned in Table 1 and the common SNPs. The outputs of SNP Clinic v.1.0 are exact transcription factors (TF) binding sequence, transcription factor binding sites (TFBS) strand (coding/+, non-coding/−), altered TFBS, a relative binding score for the Major Allele (RBSM), RBS for the Minor Allele (RBSm), affinity impact (%), homotypic redundancy (HR), Homotypic redundance Weight Factor (HWF) and the Functional Impact Factor (FIF).

### 2.3. Confirmatory Predictions for the Reported rSNPs

To evaluate the functionality of the variants predicted by SNPclinic, the scoring and prediction of the GWAVA [23] and FunSeq2 [24] tools were used. To obtain a prioritization score for functional variants, the probability score for an SNP to be an eQTL was used, using the DeepSEA [25] software.

Finally, free access databases were searched for the effect of SNPs on expression, the databases were Ensembl [26], GTEX databases [27], Expression Atlas [28], and ENCODE [29].

## 3. Results and Discussion

**Figure 1:**
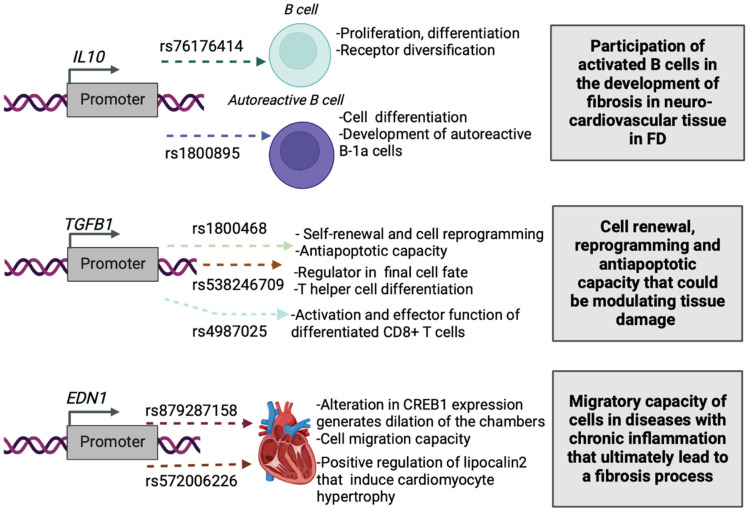
Proposed impact of the SNPClinic-predicted rSNPs in FD (“ALL” population from 1000 Genomes Project). Figure was designed in Biorender (https://biorender.com/, accessed on 15 March 2022).

In this work, the functional prediction as rSNP of 110 Common SNPs was carried out using the SNPclinic software. The classification of the true rSNPs was based on the TFBSs altered by the presence of each SNP. Through the calculated percentage of the binding affinity impact and considering the homotypic redundancy, it was obtained for the TF-SNP association, in contrast with other less sensitive methods which are mainly based on *p*-value filtering and/or ranking. Of the analyzed genes, only *IL10*, *TGFB1* and *EDN1* were obtained with the presence of rSNPs and altering TFBS (Table 2, and Figure 1).

The participation of interleukins has been seen in the pathogenic process of many diseases, within these we decided toselect the main cytokines reported to be elevated or altered in the FD process, such as TNF-α, IL-10, IL-1a, IL-1b, and IL-6. In FD it has been seen that there may be variations according to sex, and management stage such as enzyme replacement therapy that can modify interleukins levels. Serum increased levels of TNF-a have been found in women with FD. However, in men with FD, TNF-α and IL-6 are increased compared to healthy controls [31]. The proinflammatory state determined by these interleukins has also been determined by other workgroups, Biancini et al. concluded that even the levels of globotriaosylceramide (Gb3) are positively correlated with plasma levels of IL-6 [32]. The accumulation of Gb3 also has a close relationship with TNF, since TNF can increase its accumulation and be related to the pain crises present in FD, corroborating an in vitro model with peripheral blood mononuclear cells (PBMC) of male patients, who have higher expressions of TNF and IL-1β [33]. Clinical severity in FD patients, measured by the Mainz Severity Score Index (MSSI), has also been correlated with serum IL-6 and TNF-a levels, regardless of gender [31].

Of the included interleukins, we found two rSNPs in *IL10*, in the Hcm, Hac, and T_H_1 cell lines. The altered TFBS in this region were SPIB, BATF: JUN, USF2, MLX, BHLHE41, BHLHE23 and BHLHE22. Of these transcription factors it has been seen that the implication of the transcription factor SpiB is related to PU.1, together they have a participation in proliferation, survival, as well as the regulation of components of the signaling pathway of different points of differentiation of B cells, but above all, it is important to emphasize its participation in the pathways that allow the detection and response to environmental signals in B cells [34]. This could mean a key point in FD, since a decrease in fractions of memory B cells (CD20^+^/CD27^+^) has been seen in patients compared to healthy controls, but not a difference in the expression of the CD20 marker [35].

Among the other altered TFs important for differentiation are the helix-loop-helix (HLH) class, fulfilling functions of cell development and differentiation, within these USF1 and USF2 members of the bHLHZIP family are present according to the type of cell as well as their state of differentiation [36]. Other members of the basic-helix-loop-helix (bHLH) family, such as the class I bHLH proteins, E47, E12, Heb, and E2-2, interact with promoters of the pre-B-cell-specific gene for their activation, along with the activity of the early B -cell factor (EBF) [37]. Another transcription factor of the bHLH family with functions in immune cells is BHLHE41, which has been seen to participate in the control of the development of autoreactive B-1a cells, presenting in these cells a greater expression in contrast with other members of the family such as BHLHE40 [38], so this family can cover different roles in development depending on the cell.

BATF was another TFBS altered by variants in *IL10* and has been studied in various murine models, finding its key role as a regulator in class switch recombination, one of the mechanisms in which it performs this is by controlling the expression of Activation-induced cytidine deaminase (AID) [39]. AID is a member of the APOBEC family of cytidine deaminases, which facilitates receptor diversification on B cells [40]. All these TFs found by the SNPclinic support the function of IL-10 since it is known that in an autocrine manner can promote the differentiation of activated B cells, towards cells that secrete IgM and IgG [41], these TF may be part of the necessary components to fulfill this function. It is important to emphasize that we obtained these TFs in cardiac tissue and cerebellar astrocytes; at the cardiac level, the essential participation of activated B cells in the development of fibrosis after an ischemic event has already been reported, since they maintain the local inflammatory process with the expression of cytokines such as TGF-1β, IL-1β, IL-6, TNF-α and promote the expression of myocardial collagen [42]. A tissue where we mainly find altered TFBS is in the T_H_1 immunological profile that could be related to the inflammatory component and production of cytokines that the activated B cells originate, being this cell necessary for the development of neuro-cardiovascular lesions observed in FD.

Of the analyzed genes that play a role in the development of fibrosis and sclerosis, 3 rSNPs were obtained in *TGFB1*, which has been extensively studied in the development of fibrosis, participating in a feed-forward loop, where after being activated by integrins, favors the production of collagen in fibroblasts [43]. The first variant obtained was rs1800468, altering the TFBS of ESRRB, GMEB2, and CREB. ESRRB has been found functionally linked to the protein factor Ncoa3, which together can bind to RNApol2 complexes, for transcription activation, leading to cellular changes of self-renewal and reprogramming [44]. The self-renewal role of *TGFB1* has been studied for the development of different types of cancer since especially in late stages it can act as a tumor promoter [45]. The function of TF GMEB2 has been studied in conjunction with GMEB1, with a marked antiapoptotic capacity. In an in vitro study, it has been seen that the expression of GMEB-1 and GMEB-2, increase 2.82 ± 1.14 and 2.57 ± 0.31 times, respectively, after stimulating peripheral blood mononuclear cells (PBMC) with IL-12 [46]. cAMP-responsive element-binding protein (CREB) has been associated with cell proliferation and survival functions [47]. CREB overexpression can protect cells from apoptosis even in the presence of induced endoplasmic reticulum stress [48].

The relationship between the process of apoptosis and the development of fibrosis has been seen in different models, Chung et al., 2021 [49] developed a mouse model with FD, analyzing the reaction of the kidneys to profibrotic or inflammatory stimuli secondary to ureteral obstruction, found that autophagy is altered in FD, leading to renal apoptosis and fibrosis formation. There are multiple studies on the relationship between these two processes, some that support that autophagy can promote cell death and others that, on the contrary, prevent death as the destination of the cell, some authors believe that perhaps the relationship is more than the autophagy only modifies the time of cell death [50]. It has been seen that TGFB1 can increase the expression of sphingosine kinase 1 (SK1), which has been implicated in cell proliferation processes. Following SK1 activation, it can protect cells from death by inducing autophagy [51].

The second variant of *TGFB1* was rs538246709, with alteration of the TFBS for LBX1, GATA3, and ZEB1, in both Huvec and T_H_1 cell lines. LBX1 has been related as a key factor in the differentiation of embryonic dorsal neurons, called class B neurons [52], a key regulator in the transcription and final cell fate of interneurons is the Corl1 factor, acting as a co-repressor of Lbx1 [53].

LBX1 is also necessary for the correct migration of myogenic progenitors in the extremities, but this function needs the somite-derived endothelial cells, which influence the migratory behavior [54]. This could explain why we obtained rs538246709 as a putative variant in the Huvec cell line (Umbilical vein endothelial cells). The role of GATA3 has been seen particularly in key elements of T helper cell differentiation. The elimination of GATA3 in mice leads to different events in T_H_2 cells, where it produces changes in the expression of 623 genes, some specific for the immunophenotype such as Il4, Il5, Il10, Il13, and Il1rl1, on the other hand, it slightly increases T_H_1-specific genes such as Tbx21, Fasl, and Il12rb2 [55]. ZEB1 is also required in late stages for T cell differentiation, where it enhances the inhibitory effect of TGF-B1 on CD4^+^ T cells [56].

The third variant of *TGFB1* was rs4987025, with alteration of the TFBS BATF::JUN and SMAD3. A necessary component for a correct activation and effector function of differentiated CD8+ T cells is the participation of Batf::Jun, T-bet, SREBP2, and AP-1, it has been verified that they have enrichment for the mentioned TF motifs [57]. The relationship of CD8+ T cells with the rest of *TGFB1*-altered TFBS could be explained by their known interaction to perpetuate inflammation and the development of fibrosis. Their participation in the fibrotic process after influenza infection has been seen, where CD8+ resident memory T cells play a key role in the respiratory tract of aged hosts, depending on TGF-β signals [58].

Within the altered TFBS in the *TGFB1* gene promoter, we found several key elements in cell renewal, reprogramming, and antiapoptotic capacity that could ultimately be modulating tissue damage, which has been a well-studied component in FD patients. In FD nephropathy, the proximal renal tubular cell is the main producer of TGF-β 1, which initiates profibrotic changes and induces cellular differentiation of renal cells into myofibroblasts, ultimately leading to renal cell apoptosis [59]. Therefore, the altered transcription factors reported here could be essential elements to modulate the fibrotic processes that occur in FD.

Of the genes analyzed with participation in Endothelial/Vascular disease, the involvement of the Endothelin-1 gene (*EDN1)* is notable, where it induces deregulation in vasoconstriction and vasodilatation events, and has been widely seen in the literature [60,61]. The rs5370 variant of *EDN1* located in exon 5, is associated with the risk of pulmonary arterial hypertension, a disease characterized by increased pulmonary vascular resistance [62]. Due to this essential relationship in the regulation of the vessels, we analyze the promoter region, finding two variants as putatively regulatory rs879287158 and rs572006226. The TFBS for CREB1 was altered in both variants, in the cardiac myocytes, cardiac fibroblasts, astrocytes, endothelial cells, and T_H_1 cell lines, so it could be fulfilling an essential role in the function of *EDN1*. The participation of the TF cyclic AMP response element (CRE)-binding protein (CREB) has been seen in models of transgenic mice, when a dominant-negative expression of this TF occurs, generates dilation of the chambers and thinning of ventricular walls, reflecting features of dilated cardiomyopathy [63]. It is known that the distribution of expression in cardiac tissue of the members of the CREB family is different according to the type of cell, where there is an expression of CREB exclusively in fibroblasts and of CREM in myocytes [64]. Interestingly, we found this altered TF with FIF score without any difference between myocytes and cardiac fibroblasts.

There is also a relationship between *EDN1* with the formation of dilated cardiomyopathy, in murine models with an expression of Edn1 that progressively decreases, produces a deterioration of cardiac function, with increased plasma volumes, and finally causes dilated cardiomyopathy [65]. Therefore, CREB1 could be directly influenced in the development of dilated cardiomyopathy already reported for *EDN1*. In Fabry disease, the main cardiac manifestation is left ventricular hypertrophy (LVH) [66], although there are also reports of manifestations such as congestive heart failure such as dilated cardiomyopathy [67].

Another of the transcription factors that we found altered in different cell lines was TCF3, with changes in both cardiac fibroblasts and endothelial cells. TCF3 has been related to cell migration capacity, this effect is only seen by TFC4 and not by the rest of the members of the Lef/Tcf family. The mechanisms by which it induces this effect are due to the positive regulation of lipocalin2 (LCN2) [68]. LCN2, on the other hand, can induce cardiomyocyte hypertrophy. When it is overexpressed, it can even decrease the number of cells and reduce cell mitosis in cardiac tissue [69]. The function of LCN2 has also been seen to induce fibrosis, mainly in human alcoholic hepatitis, where overexpression correlates with portal hypertension and the degree of fibrosis. Specifically, in human hepatic stellate cells, LCN2 can increase the expression of *EDN1* mediated by HIF1A [70].

Participation in cell migration by *EDN1* was confirmed in a model of hepatocarcinogenesis, which is a process that evolves from inflammation, cirrhosis, fibrosis, to finally, liver carcinoma. *EDN1* can stimulate the expression of cell cycle genes, cell proliferation, and migration, these functions may be mediated by the activation of the AKT signaling pathway [71]. The migratory capacity of cells could be playing an essential role in diseases with chronic inflammation that ultimately lead to a fibrosis process, with TCF3 perhaps being the preliminary component for the development of this process. Regarding the rest of the altered TFBS, NOTO, HOXD12, and USF1, we did not find a relationship with the cellular migratory capacity or in the fibrosis process.

The rest of the genes analyzed, interleukins, the participants in fibrosis processes, vascular and renal disease, without rSNP results, could be due to the lack of relevance in the cell lines affected by FD, or because they could be having a secondary regulation.

Of the rSNPs proposed by SNPclinic, we corroborate it with other software such as GWAVA, where we find similar results, with a final prediction of deleterious, in 70% of the rSNPs. In addition, to corroborate not only its classification as a deleterious variant but also the regulation in the expression, the probability score of being an eQTL was obtained for each variant, obtaining a score equivalent to that given by the other tools. Finally, the effect of the variants in different tissues was evaluated, see Table 3.

The participation of the genes analyzed in this article reflects the growing interest in the study of variants in modifying genes, which can influence as enhancers or suppressors of the severity of diseases and in most cases may not result in any phenotype by themselves [72]. Gaucher disease (GD) has been an example where it has been continuously analyzed how modifying genes, epigenetics, and other factors can lose the clear limits between simple and complex inheritance [73]. Durán et al., 2021 found 271 Single Nucleotide Variants (SNVs) within nine genes associated with the hepatic activity of the enzyme β-glucocerebrosidase that causes GD, which could act as modifying the activity of the enzyme [74]. In Charcot-Marie-tooth disease type 1A (CMT1A), mutations in the *LITAF* gene can predispose to the appearance of CMT1A for up to 13 years, in contrast with those who do not present the variant [75]. Other genes have also been associated with CMT1A, such as *SIPA1L2*, which may increase the severity of anterior tibial muscle weakness, leading to foot drop [76]. In other archetypal monogenic disorders, a great clinical heterogeneity has been seen, as is the case of β-thalassemia, in which factors can even improve the clinical phenotype, as is the case of variants in the *BCL11A* gene [77]. Some groups have focused on the importance of genetic background, in diseases such as PMM2-CDG the most common disorder of glycosylation (CDG), finding possible disease-modifying genes [78]. One problem facing the identification of genetic modifiers is that the vast majority are based on small portions of the phenotypic variability, such as individual families [79]. We propose to consider the component of modifier genes in the clinical presentation of FD, similar to what has already been proposed for other diseases, so that the *IL10*, *TGFB1*, and *EDN1* genes could explain part of the phenotypic heterogeneity.

## 4. Conclusions

In conclusion, we found seven rSNPs in three genes that could partially explain the variations in the phenotype of patients with FD, these are involved in processes such as proliferation, survival, and state of differentiation of B cells. Once activated, B cells could favor chronic inflammation that leads to fibrosis. Fibrosis is an essential component in the apoptosis-autophagy regulation axis.

The rSNPs proposed here could modulate the clinical phenotype of Fabry disease, so we propose that *IL10*, *TGFB1* and *EDN1* genes be considered modifier/minor genes in FD, in charge of regulating its neuro-cardiovascular variant.

## Figures and Tables

**Table 1:** List of the genes and their total number of common SNPs in proximal promoters that were analyzed with SNPClinic v.1.0 software.

Selection Criteria	Gene	Common SNPs
Interleukins	*TNF*	*3*
	*IL10*	*8*
	*IL1A*	*6*
	*IL1B*	*5*
	*IL6*	*7*
Fibrosis and sclerosis	*TGFB1*	*9*
	*FGF2*	*11*
	*MMP1*	*9*
Renal disease	*REN*	*14*
	*AGTR1*	*8*
	*AGT*	*3*
Endothelial/Vascular disease	*EDN1*	*8*
	*NOS3*	*10*
	*MTHFR*	*9*
TOTAL	*14*	*110*

**Table 2:** Prediction of rSNPs with SNPClinic v.1.0 software. SNPClinic outputs include cell line specificity, altered TFBS and quantitative ranking according to transcriptional relevance with the Functional Impact Factor (FIF).

Gene	rSNP	Chromatin-Accesible Cell Line ^1^	Transcription Factor	RBS_M_	RBSm	Affinity Impact%	HR	HWF	FIF ^2^
***IL10***	rs76176414	Hcm	SPIB	0.87	0.70	−19.57	1	1	−19.57
		Hac	BATF:JUN	0.82	0.72	−11.54	1	1	−11.54
	rs1800895	T_H_1	USF2	0.94	0.81	−13.27	1	1	−13.27
			MLX	0.80	0.68	−15.10	1	1	−15.10
			BHLHE41	0.81	0.70	−13.42	1	1	−13.42
			BHLHE23	0.87	0.96	10.40	1	1	10.40
			BHLHE22	0.86	0.96	11.51	1	1	11.51
***TGFB1***	rs1800468	Hcm	ESRRB	0.84	0.73	−12.77	1	1	−12.77
			GMEB2	0.83	0.66	−20.76	1	1	−20.76
			CREB1	0.86	0.73	−15.32	1	1	−15.32
	rs538246709	Huvec	LBX1	0.80	0.67	−16.15	1	1	−16.15
			GATA3	0.86	0.73	−14.65	1	1	−14.65
			ZEB1	0.83	0.69	−16.54	1	1	−16.54
	rs4987025	Huvec	BATF::JUN	0.80	0.68	−14.20	1	1	14.20
			SMAD3	0.80	0.70	12.52	1	1	−12.52
	rs538246709	T_H_1	LBX1	0.80	0.67	−16.15	1	1	−16.15
			GATA3	0.86	0.73	−14.65	1	1	−14.65
			ZEB1	0.83	0.69	−16.54	1	1	−16.54
***EDN1***	rs879287158	T_H_1	CREB1	0.80	0.93	16.53	1	1	16.53
			NOTO	0.80	0.61	−23.68	2	0.5	−11.84
	rs572006226	Hac	HOXD12	0.81	0.69	−14.93	1	1	−14.93
	rs879287158	Hcf	CREB1	0.80	0.93	16.53	1	1	16.53
			TCF3	0.82	0.65	−20.09	2	0.5	−10.04
		Hcm	CREB1	0.80	0.93	16.53	1	1	16.53
		Huvec	USF1	0.81	0.69	−15.61	1	1	−15.61
			CREB1	0.80	0.93	16.53	1	1	16.53
			TCF3	0.82	0.65	−20.09	2	0.5	−10.04

^1^. Chromatin-accesible cell line: Hcm (Human Cardiac Myocytes), Hcf (primary Human Cardiac Fibroblasts), Hac (Astrocytes-cerebellar), Huvec (Umbilical vein endothelial cells), T_H_1. Binding score for the Major Allele (RBS_M_), RBS for the Minor Allele (RBSm), affinity impact (%), homotypic redundance (HR), homotypic redundance weight factor (HWF) and functional impact factor (FIF), Ruiz Ramírez et al. [30]. ^2^. Negative values in FIF indicate a prediction of decreased affinity between TF-DNA of minor allele compared with major allele. Positive values in FIF indicate a prediction of increased affinity between TF-DNA of minor allele compared with major allele. Positive values of FIF >10 suggest the creation of a new TFBS.

**Table 3:** Functional confirmation of putative rSNPs in other databases, first the classification of variants was carried out using the tools GWAVA and Funseq2, secondly, a score was obtained in DeepSea, for each SNP the probability of contributing to the expression as eQTL was found. Finally, the effect of each SNP on tissue-dependent expression was obtained in the ENSEMBL, GTEx, EBI-EMBL and ENCODE databases. Confirmed true rSNPs (firstly predicted by SNPclinic v.1.0) are shown in bold numbers.

*GENE*	rSNP	GWAVA Score/Prediction	FunSeq2 Score/Prediction	DeepSea eQTL Probability	SNPClinic/ENCODE Cell Lines	ENSEMBLEffect Size ^1^	GTExm-Value ^2^	EBI-EMBL Expression Atlas (TPM/FPKM) ^3^	ENCODE (TPM/FPKM + 0.01) ^4^
*IL10*	rs76176414	0.32/neutral	ND	**0.68**	Hcm	ND	ND	**0.7**	**0.01**
					Hac	ND	ND	0.4	**0.01**
	rs1800895	0.33/neutral	0/neutral	**0.70**	T_H_1	**0.22**	ND	**0.9**	**0.37**
*TGFB1*	rs1800468	**0.78/deleterious**	0.49/neutral	**0.64**	Hcm	**0.24**	ND	**27**	**8.05**
	rs538246709	**0.58/deleterious**	0.49/neutral	**0.55**	Huvec	ND	ND	**145**	**7.25**
	rs4987025	**0.55/deleterious**	0.49/neutral	**0.57**	Huvec	ND	ND	**145**	**7.25**
	rs538246709	**0.58/deleterious**	0.49/neutral	**0.55**	T_H_1	ND	ND	**38**	**4.93**
*EDN1*	rs879287158	**0.71/deleterious**	**3.22/deleterious**	**0.95**	T_H_1	ND	ND	**2**	**1.04**
	rs572006226	**0.47/deleterious**	**3.59/deleterious**	ND	Hac	ND	ND	**6**	**5.85**
	rs879287158	**0.71/deleterious**	**3.22/deleterious**	**0.95**	Hcf	ND	ND	**8**	**2.05**
					Hcm	ND	ND	**8**	**1.18**
					Huvec	ND	ND	**557**	**5.69**

^1^. Effect size: Effect of the alternative allele (ALT) relative to the reference allele (REF). The eQTL effect allele is the ALT allele. ^2^. m-value:<0.1 the tissue/cell line is predicted to not have an eQTL effect; >0.9: the tissue/cell line is predicted to have an eQTL effect. ^3^. TPM/FPKM 0.5: expression level is below cutoff; TPM/FPKM from 0.5 to 10: expression level is low. TPM/FPKM from 11 to 1000: expression level is medium. More than 1000 TPM or FPKM: expression level is high. ^4^. Gene Expression Profiles by RNA-seq presented in log^2^ (TPM/FPKM + 0.01). ND: No data available.

## Data Availability

1. The datasets analyzed during the current study are available in the Ensembl Genome Browser 102, 2020. https://www.ensembl.org/index.html. Accessed: 15 March 2022. 2. The datasets analyzed during the current study are available in the Genotype-Tissue Expression (GTEx) Consortium, GTEx Project, 2021. https://gtexportal.org/home/GTEx/. Accessed: 15 March 2022. 3. The datasets analyzed during the current study are available in the European Molecular Biology Laboratory-European Bio-informatics Institute, Gene Expression Atlas, 2021. https://www.ebi.ac.uk/gxa/home. Accessed: 15 March 2022. 4. The datasets analyzed during the current study are available in the ENCODE portal. https://www.encodeproject.org. Accessed: 15 March 2022.
